# Hemispheric Bases for Emotion and Memory

**DOI:** 10.3389/fnhum.2014.00997

**Published:** 2014-12-05

**Authors:** Tad T. Brunyé, Sarah R. Cavanagh, Ruth E. Propper

**Affiliations:** ^1^Department of Psychology, Tufts University, Medford, MA, USA; ^2^Cognitive Sciences, U.S. Army Natick Soldier Research, Development and Engineering Center (NSRDEC), Natick, MA, USA; ^3^Department of Psychology, Assumption College, Worcester, MA, USA; ^4^Department of Psychology, Montclair State University, Montclair, NJ, USA

**Keywords:** emotion, brain, memory, laterality, hemispheres

The goal of this Research Topic was to bring together diverse scientific perspectives on lateralized brain mechanisms underlying emotion, motivation, and memory. The Topic resulted in eight articles, three of which report original research and five of which review and synthesize past research with the aim of developing new hypotheses and theory. A range of international experts with diverse backgrounds, theoretical perspectives, and experimental methods contributed to the Topic. Contributions strongly reflect this diversity, ranging from examining pupil dilation in response to viewing Rembrandt portraits to understanding how caffeine supplementation influences levels of spatial processing. In all cases, the authors developed strong, empirically guided insights into the lateralized brain mechanisms underlying behavioral effects. Two primary themes emerge to guide and constrain continuing research.

The first theme is related to *dynamic interhemispheric interactions* that subserve emotion, motivational states, and memory. Elizabeth Shobe’s article, *Independent and Collaborative Contributions of the Cerebral Hemispheres to Emotional Processing* (Shobe, 2014) proposes a framework for understanding the interaction of lateralized brain mechanisms for identifying and understanding emotional stimuli and engaging in higher-order emotional processing. Under this framework, the right hemisphere engages subcortical structures with the goal of identifying and comprehending positive and negative emotional stimuli, whereas the left hemisphere contributes to higher-level processing such as emotion regulation and adaptation. Critically, dynamic interhemispheric interactions provide the left hemisphere with the information it needs to execute relatively strategic processes. Spielberg and colleagues emphasize the importance of lateralized approach versus avoidance networks in guiding human behavior. In their article, *Hierarchical Brain Networks Active in Approach and Avoidance Goal Pursuit* (Spielberg et al., 2013), the authors propose a hierarchical model consisting of four levels: tactical, strategic, system, and temperamental, following a neurally inspired abstraction gradient along posterior to anterior areas of the prefrontal cortex. Right hemisphere regions process and update avoidance goals, and left hemisphere regions govern approach goals. The model dictates both intrahemispheric interactions across hierarchical layers, and also interhemispheric interactions that cut across both abstraction levels and motivational states; together, these interactions guide, constrain, and update goal-directed behavior over time.

Two particular original research contributions also fit the theme related to interhemispheric interaction. The first is Parker and colleagues’ *Effects of Saccadic Bilateral Eye Movements on Episodic and Semantic Autobiographical Memory Fluency* (Parker et al., 2013), demonstrating that horizontal saccadic eye movements enhance episodic but not semantic autobiographical memory retrieval. In accounting for these results, the authors point to the hemispheric encoding/retrieval asymmetry (HERA; Habib et al., 2003) model and its suggestion that episodic memory retrieval depends on efficient and dynamic interhemispheric interactions. They also suggest that saccadic eye movements may induce transient increases in executive function that serve to direct attention toward recalling specific episodic details, which is a unique proposition that will likely motivate subsequent research expanding the nature and scope of dependent measures used in these types of experiments. The second original research contribution fitting the theme of interhemispheric interaction is provided by Edlin and colleagues in their article *Memory for Hand-use Depends on Consistency of Handedness* (Edlin et al., 2013), demonstrating that inconsistent handedness enhances episodic memory for manual actions. Inconsistent-handers more frequently engage bilateral motor regions of the brain, and also have larger corpora callosa relative to consistent-handers, suggesting that increased hemispheric interaction may underlie these reported effects.

The second theme that emerged from this Research Topic is *identifying and characterizing lateralized processes* in both human beings and non-human primates. In this theme, very diverse contributions were made characterizing the universality of lateralized emotion perception and expression across primates on the one and hand, and artistically, nutritionally, and clinically altering levels of lateralized task engagement on the other. Lindell contributed a review article, *Continuities in Emotion Lateralization in Human and Non-human Primates* (Lindell, 2013), providing compelling evidence that both human beings and non-human primates, including rhesus macaques and chimpanzees, use primarily right hemisphere brain mechanisms for generating and perceiving facial emotions, suggesting some universality of lateralized emotional expression and perception. Two other reviews examined lateralized contributions to perception and memory. First, Willment and Golby (2013) support a material-specific model of how focal pathology and surgical management of temporal lobe epilepsy can result in predictable memory deficits, with left and right temporal regions mediating verbal and non-verbal memories, respectively. Such material-specific links can inform preoperative fMRI memory mapping aimed at predicting and managing post-operative memory outcomes. This is an exciting prospect, and demonstrates a promising avenue for transitioning research on lateralized emotion and memory to high stakes clinical settings. Second, Schirillo (2013) demonstrates that viewing male Rembrandt portraits reliably produced pupil dilations when the portraits were rated as low or high in valence. The author suggests that right lateralized brain mechanisms responsible for emotion perception may explain why some artists may choose to depict the right cheek of male figures, but the left cheek of female figures, perhaps promoting perceptions of dominance in male figures. The final contribution related to identifying and characterizing lateralized processes was provided by Giles and colleagues in their article *Caffeine Promotes Global Spatial Processing in Habitual and Non-habitual Caffeine Consumers* (Giles et al., 2013). The authors found that increasing doses of caffeine promoted memory for distal but not proximal landmark relationships during a spatial memory test. They suggest that such global enhancement may be related to caffeine-induced upregulation of brain dopamine, serotonin, and norepinephrine, neurotransmitter systems that may be higher density in right versus left hemisphere brain regions.

The diverse articles published under this Research Topic advance theoretical positions related to interactive contributions of brain hemispheres toward a broad range of cognitive functions related to emotion and memory, and highlight important characteristics of lateralized mechanisms in human and non-human primates. Relatively, integrated perspectives on brain involvement across a range of cognitive tasks provide stimulating theoretical frameworks for reconciling a range of experimental findings and motivating future research. Specific outstanding questions raised by the Research Topic include:

(1) How might other individual differences modulate lateralized and bilaterally interactive brain mechanisms involved in processing specialized information types and maintaining and updating motivational states and goals?
(2) Contrasting accounts of hemispheric differences in affective and motivational processing were presented. How can we devise research paradigms to test the relative validity of these models?
(3) Much of this work infers lateralized processes from behavior, but how might functional connectivity analyses advance our understanding, for instance by exploring interhemispheric communication as a result of saccadic eye movements or inconsistent handedness?
(4) How might a wider range of nutritional influences, including psychostimulants and amino acids, bias attention, perception, and memory toward particular information types, motivational states, arousal, and mood states, or levels of focus?
(5) How can better understanding lateralized and bilateral interactive brain processes inform applied interventions for clinical or performance-based domains, including predicting surgical outcomes, and informing targeted neuromodulation (i.e., TMS, tDCS)?

## Conflict of Interest Statement

The authors declare that the research was conducted in the absence of any commercial or financial relationships that could be construed as a potential conflict of interest.
